# Assessment of Knowledge-Based Planning for Prostate Intensity Modulated Proton Therapy

**DOI:** 10.14338/IJPT-20-00088.1

**Published:** 2021-06-15

**Authors:** Yihang Xu, Nellie Brovold, Jonathan Cyriac, Elizabeth Bossart, Kyle Padgett, Michael Butkus, Tejan Diwanj, Adam King, Alan Dal Pra, Matt Abramowitz, Alan Pollack, Nesrin Dogan

**Affiliations:** 1 Department of Radiation Oncology, University of Miami Miller School of Medicine, Miami, FL, USA; 2 Department of Radiation Oncology, Henry Ford Health System, Detroit, MI, USA

**Keywords:** knowledge-based planning, IMPT, prostate cancer

## Abstract

**Purpose:**

To assess the performance of a proton-specific knowledge based planning (KBPP) model in creation of robustly optimized intensity-modulated proton therapy (IMPT) plans for treatment of patients with prostate cancer.

**Materials and Methods:**

Forty-five patients with localized prostate cancer, who had previously been treated with volumetric modulated arc therapy, were selected and replanned with robustly optimized IMPT. A KBPP model was generated from the results of 30 of the patients, and the remaining 15 patient results were used for validation. The KBPP model quality and accuracy were evaluated with the model-provided organ-at-risk regression plots and metrics. The KBPP quality was also assessed through comparison of expert and KBPP-generated IMPT plans for target coverage and organ-at-risk sparing.

**Results:**

The resulting *R*^2^ (mean ± SD, 0.87 ± 0.07) between dosimetric and geometric features, as well as the χ^2^ test (1.17 ± 0.07) between the original and estimated data, showed the model had good quality. All the KBPP plans were clinically acceptable. Compared with the expert plans, the KBPP plans had marginally higher dose-volume indices for the rectum V65Gy (0.8% ± 2.94%), but delivered a lower dose to the bladder (−1.06% ± 2.9% for bladder V65Gy). In addition, KBPP plans achieved lower hotspot (−0.67Gy ± 2.17Gy) and lower integral dose (−0.09Gy ± 0.3Gy) than the expert plans did. Moreover, the KBPP generated better plans that demonstrated slightly greater clinical target volume V95 (0.1% ± 0.68%) and lower homogeneity index (−1.13 ± 2.34).

**Conclusions:**

The results demonstrated that robustly optimized IMPT plans created by the KBPP model are of high quality and are comparable to expert plans. Furthermore, the KBPP model can generate more-robust and more-homogenous plans compared with those of expert plans. More studies need to be done for the validation of the proton KBPP model at more-complicated treatment sites.

## Introduction

Prostate cancer is the second most-frequent cause of cancer death among men in the United States and the leading cause of cancer death among men in 46 countries [1]. Intensity-modulated radiation therapy (IMRT), volumetric-modulated arc therapy (VMAT), and intensity-modulated proton therapy (IMPT), which can deliver a highly conformal dose to the tumor and spare organs at risk (OARs), are advanced radiation-therapy techniques commonly used for treatment of prostate cancer [2, 3]. Because of the physical property of proton beams that can eliminate the “exit dose” beyond the Bragg peak, proton therapy has the potential to improve the target coverage and provide better OAR sparing compared with photon-based radiation therapy. Several publications have shown that IMPT can deliver superior dose distributions compared with IMRT/VMAT for the treatment of prostate cancer [4, 5]. Similar to IMRT, IMPT uses inverse-planning optimization to achieve dosimetric objectives. However, the complexity of IMPT planning, combined with differences in the experience and skill of the planners, may result in large variations in the quality of treatment plans, leading to suboptimal dose distribution [6–8].

Knowledge-based planning (KBP) tools, which incorporate prior treatment planning experience, have the potential to improve the quality and consistency of treatment plans [9–13]. Numerous studies have demonstrated that KBP models are able to generate IMRT and VMAT plans comparable to, or even better than, expert plans for a range of treatment sites [14–19]. Recently, a proton-specific KBP (KBPP) was developed to accommodate the physical traits of protons (eg, no dose beyond the Bragg Peak) into the dose-volume histogram (DVH) estimation model [20]. A few publications have explored the usefulness of the KBPP. Delaney et al [21] originally illustrated the concept of applying the knowledge-based DVH estimation model to select patients (before starting optimization) to help determine those that would benefit greatly from proton therapy, as compared to VMAT photon therapy, for patients with head and neck cancer. Their later publication demonstrated that clinically acceptable IMPT plans can be created using a KBPP system [20, 22]. A recently published study by Cozzi et al [23] showed that the quality of the KBPP plans were at least equivalent to the manually generated expert plans for patients with hepatocellular carcinoma. However, more studies are necessary to both evaluate and validate the KBPP for various treatment sites at this early stage in its use. To our knowledge, there are no studies investigating the use of KBPP for IMPT planning of prostate cancer. In this work, we assessed the performance of a KBPP model in the creation of robustly optimized IMPT plans for patients with prostate cancer.

## Materials and Methods

### Patient Cohort and IMPT Planning

Forty-five patients with localized prostate cancer who were previously treated with VMAT and were enrolled on a prospective institutional review board protocol were included in this study. Patients were scanned for treatment planning in supine position with 1.5-mm-slice thickness and had both a full bladder and an empty rectum per local protocol [24]. For all patients, the prostate gross tumor volume, clinical target volume (CTV), bladder, rectum, and femoral heads were delineated on the planning computed tomography (CT) scan by a radiation oncologist. The CTV consisted of prostate and proximal seminal vesicles.

Before the planning CT scan, patients were prepared with bladder and rectal preparation protocol to comfortably fill the bladder and release the gas inside the rectum. If gas was still detected inside the rectum in the planning CT, it was overridden to water-equivalent density. Streaking artifacts produced by gold fiducials were also overridden to the surrounding soft-tissue density for proton planning. Thirty IMPT plans were generated by an experienced proton dosimetrist for model configuration. These IMPT plans employed 2 opposed lateral fields with a multifield optimization (MFO) technique using the nonlinear universal proton optimizer (NUPO version 15.6, Eclipse, Varian Medical Systems, Palo Alto, California). Dose calculation was performed using the proton convolution superposition algorithm (PCS version 15.6, Eclipse, Varian). A relative biological effectiveness (RBE) of 1.1 is used for representation of the RBE-weighted dose. The prescription dose to the CTV was 78 to 80 Gy (RBE) in 38 to 40 fractions. Plans were robustly optimized using ± 5-mm setup uncertainty (in cardinal directions), along with ± 3% proton-range uncertainty. The dose constraints for CTV were V100 (relative volume receiving more than the prescription dose) > 99.99% and *D*_max_ (maximum relative dose delivered to the structure) < 115%. The dose constraints for the rectum were V40Gy (relative volume of the structure receiving > 40 Gy) < 35%, V65Gy (relative volume of the structure receiving > 65 Gy) < 17%, and V80Gy (relative volume of the structure receiving > 80 Gy) < 10%, whereas for the bladder, the constraints were V40Gy < 50%, V65Gy < 25%, and V80Gy < 10%. The dosimetrist made an effort to keep OAR doses (bladder and rectum) as low as possible. The plan evaluations were then performed for the finalized plans by simulating the ± 3% proton-range uncertainty with ± 5-mm translational error in 6 directions, resulting in 12 uncertainty scenarios. In the robust evaluation, the worst-case scenario was required to achieve V95 > 95% (≥ 95% of the volume receiving > 95% of the prescription dose) for CTV.

### KBP Model Configuration

A KBPP-optimization tool (RapidPlanPT, version 16.1, Varian) was used to create the KBP library. RapidPlanPT consists of 2 phases for model configuration: the data-extraction phase and the model-training phase. In the data-extraction phase, the geometric and dosimetric features of selected structures are parameterized for use in model training. During the model-training phase, the DVH-estimation algorithm is applied to create a DVH-estimation model. Individual structure objectives and priorities may be set or generated based on the training set and their principal components. As described in Delaney et al [20], RapidPlanPT incorporates a simplified spread-out Bragg peak into the model and uses the geometry-based expected dose metric to estimate the distance of the different voxels in each structure from the target surfaces. Delaney et al [20] have described RapidPlanPT modeling in greater detail as well as the differences between the photon-based model and the proton-based model in their work, so these details will not be included in this work.

In our study, 30 IMPT plans created using the MFO technique for patients with prostate cancer were included in the proton RapidPlan model library. A defined objective list was implemented in the model after initial model training. The priority for each objective was set to automatically generate optimization for prospective patients. The model quality was assessed using model-generated plots, such as DVH plots, and regression and residual plots, based on the principal-component analysis, as well as some additional metrics to identify potential geometric and dosimetric outliers [25]. Coefficient of determination (*R*^2^) and average Chi-square (χ^2^) tests were applied to measure the goodness of fit of the model for each trained OAR, where the *R*^2^ indicated the correlation between dosimetric and geometric features, whereas χ^2^ represented the difference between the original and the estimated data [25].

### Model Validation

The remaining 15 patients who were not included in the model training served as the model-validation group. For each patient used for model validation, both MFO-based expert and KBPP-generated plans were created. The KBPP plans used the same beam arrangement as the corresponding expert plans. For comparison purposes, the expert plans, with single-field optimization (SFO) technique for 10 validation cases, were also created and compared with the corresponding KBPP-generated plans using the same technique.

The KBPP plans were assessed and compared with the expert plans using the same clinical dose volume constraints for CTV, bladder, and rectum. In addition, we assessed the integral dose deposited in the structure, which removed the CTV volume extending 1 cm outside from the external volume contour (*External* − [*CTV* + 1 cm]). The homogeneity index (*HI*) was also evaluated for KBP-based IMPT plans and compared with that of the expert plans. In this work, the *HI* was defined as follows [26]:
(1)

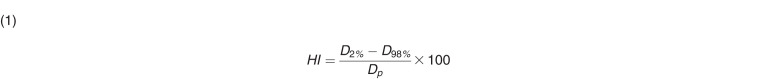
where *D*_2%_ is the dose to 2% of the CTV, *D*_98%_ is the dose to 98% of the CTV, and *D_p_* is the prescription dose for the CTV. The closer the *HI* value is to zero, the more homogenous the plan is. Both expert- and KBP-generated IMPT plans were normalized, such that 99.99% of the CTV volume was covered by the 100% prescription dose. To analyze the robustness of the IMPT plan, all uncertainty scenarios and nominal plan generated from both expert and KBP plans were included for the comparison. The comparison of the dosimetric indices between KBPP and expert plans was performed with a 2-sided paired *t* test. *P* < .05 was considered statistically significant.

## Results

### Model-Training Results

**Figure 1.**
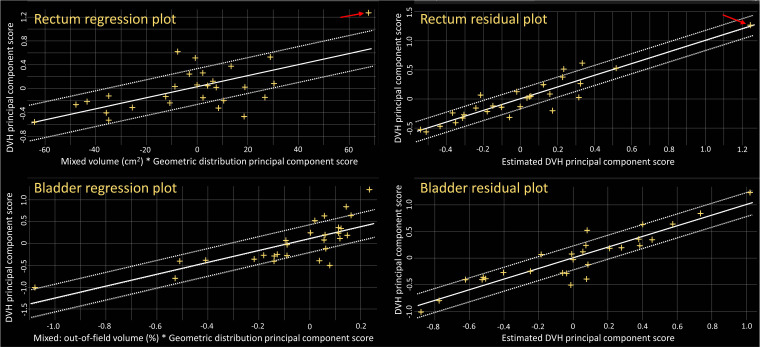
Regression plots and residual plot for rectum and bladder

Rectum, bladder, bowel, left and right femoral heads, penile bulb, and urethra were included in the model training. The *R*^2^ (mean ± SD, 0.87 ± 0.07, range, 0.79–0.99) and χ^2^ (1.17 ± 0.07, 1.09-1.29) for each OAR indicates good quality of the model. Two potential outliers for the rectum and 3 potential outliers for the bladder were identified according to metrics in the final model report. **Figure 1** shows the regression plots and residual plots for the rectum and bladder. The regression plots indicate the correlation between the most-important geometric-regression parameter and the main DVH parameter, which can be used for potential geometric-outlier identification. The residual plots evaluate how the original DVH of a structure differ from the estimated DVH, and they were used as a more-realistic evaluation of potential influential points that can significantly affect the outcome of the DVH-estimation model. Although some of the points are greater than the confidence interval in the residual plots, we think none of these points is a significant influential point because no point deviated greatly from the fitting line isolated by itself. In the case of the isolated points (arrowed in the plots) indicated in **Figure 1**, the rectum was extremely large and a greater percentage of volume was on the proton path compared with that of other cases, which was deemed an acceptable geometric outlier in the context of the data. Based on the resulting *R*^2^, χ^2^, and visual verification of the plots, we decided not to exclude any other outliers. This finding is consistent with previous studies, which also showed that the removal of outliers from a good-quality KBP model library with a sufficient population does not have a significant effect on the quality of the plans [15, 27]

### Comparison between KBPP Plans and Expert Plans

All plans generated by the KBPP were clinically acceptable, and most plans met the dose-volume constraints, except for 1 case with very small bladder, which will be discussed later. The **Table** shows the comparison of dosimetric indices between the KBPP-generated and the expert plans, based on both the MFO and SFO techniques. For MFO-based plans, KBPP plans had marginally higher dose-volume indices for the rectum V65Gy (0.8% ± 2.94%) but had lower values for all clinical bladder dose volume indices (−1.06% ± 2.9% for bladder V65Gy). In addition, the KBPP plans achieved lower hot spots (−0.67 ± 2.17 Gy) and lower integral doses (−0.09 ± 0.3 Gy) than the expert plans did. Moreover, the better KBPP-generated plans demonstrated slightly greater CTV V95 (0.1% ± 0.68%) and lower *HI* (−1.13 ± 2.34). For SFO-based plans, there is no statistically significant difference for CTV coverage and *HI*. Nevertheless, KBPP plans based on SFO had better rectum sparing than the expert plan had, but they had higher bladder dose-volume indices and higher hot spots. The *D*_max_, in the structure *External* − (CTV + 1 cm), of KBPP plans was hotter than that of the expert plans. Because the purpose of this work was to compare KBPP plans against the expert plans, hereafter, we focus only on the results using the MFO technique because the model was configured with all MFO-based plans.

**Table.** Comparison between KBPP and expert plans for both MFO- and SFO-based plans. All uncertainty scenarios and the nominal plan were included for the dose mean and standard deviation calculations. The same scenarios were compared with the differences calculated. Note that MFO plans were generated for validation with 15 patients, whereas SFO plans were generated for 10 patients.

**Dose-volume indices**	**MFO, n = 15**				**SFO, n = 10**			
	**Expert, mean ±SD**	**KBPP, mean ±SD**	**KBPP-expert, mean ±SD**	***P*** **value**	**Expert, mean ±SD**	**KBPP, mean ±SD**	**KBPP-Expert, mean ±SD**	***P*** **value**
CTV V95, %	99.09 ± 1.02	99.19 ± 0.91	0.1 ± 0.68	**0.039**	99.09 ± 0.95	99.2 ± 1.02	0.11 ± 0.72	0.088
CTV D99, Gy	74.71 ± 4.05	75.09 ± 4	0.39 ± 2.12	**0.012**	75.32 ± 4.28	75.43 ± 4.61	0.11 ± 2.2	0.594
CTV *HI*	8.61 ± 3.49	7.47 ± 3.2	−1.13 ± 2.34	**<0.001**	6.2 ± 3.27	6.22 ± 3.78	0.02 ± 2.2	0.93
Rectum V40Gy, %	13.82 ± 9.02	14.29 ± 9.44	0.47 ± 4.47	0.142	12.89 ± 9.17	11.45 ± 7.2	−1.44 ± 3.74	**<0.001**
Rectum V65Gy, %	6.03 ± 5.73	6.83 ± 6.28	0.8 ± 2.94	**<0.001**	5.58 ± 5.66	4.79 ± 4.55	−0.78 ± 2.34	**<0.001**
Rectum V80Gy, %	1.19 ± 2.18	1.22 ± 2.28	0.04 ± 1.16	0.641	0.86 ± 1.92	0.88 ± 1.94	0.02 ± 0.32	0.534
Bladder V40Gy, %	15.65 ± 13.89	13.81 ± 12.52	−1.84 ± 4.29	**<0.001**	13.1 ± 14.1	13.87 ± 14.75	0.77 ± 1.5	**<0.001**
Bladder V65Gy, %	8.26 ± 9.45	7.19 ± 8.1	−1.06 ± 2.9	**<0.001**	6.76 ± 8.93	7.55 ± 9.99	0.79 ± 1.64	**<0.001**
Bladder V80Gy, %	1.62 ± 3.27	1.56 ± 3	−0.06 ± 1.59	0.618	1.2 ± 2.49	1.45 ± 2.98	0.26 ± 0.67	**<0.001**
*D*_max_, Gy	85.45 ± 2.88	84.79 ± 3.01	−0.67 ± 2.17	**<0.001**	82.65 ± 3.37	84.58 ± 4.38	1.93 ± 1.63	**<0.001**
*External* − (CTV + 1 cm), *D*_mean_, Gy	1.2 ± 0.45	1.11 ± 0.3	−0.09 ± 0.3	**<0.001**	1.09 ± 0.3	1.09 ± 0.31	0 ± 0.04	0.077
*External* − (CTV + 1 cm), *D*_max_, Gy	78.67 ± 5.15	77.99 ± 4.6	−0.68 ± 2.9	**0.001**	76.61 ± 4.27	79.66 ± 5.48	3.05 ± 2.66	**<0.001**

Abbreviations: KBPP, proton-specific knowledge-based planning; MFO, multifield optimization; SFO, single-field optimization; *HI*, homogeneity index; VxxGy, relative volume of the structure receiving > xx Gy; *D*_max_, maximum relative dose delivered to the structure.

Note: Bolded values were considered statistically significant at *P* < .05.

**Figure 2.**
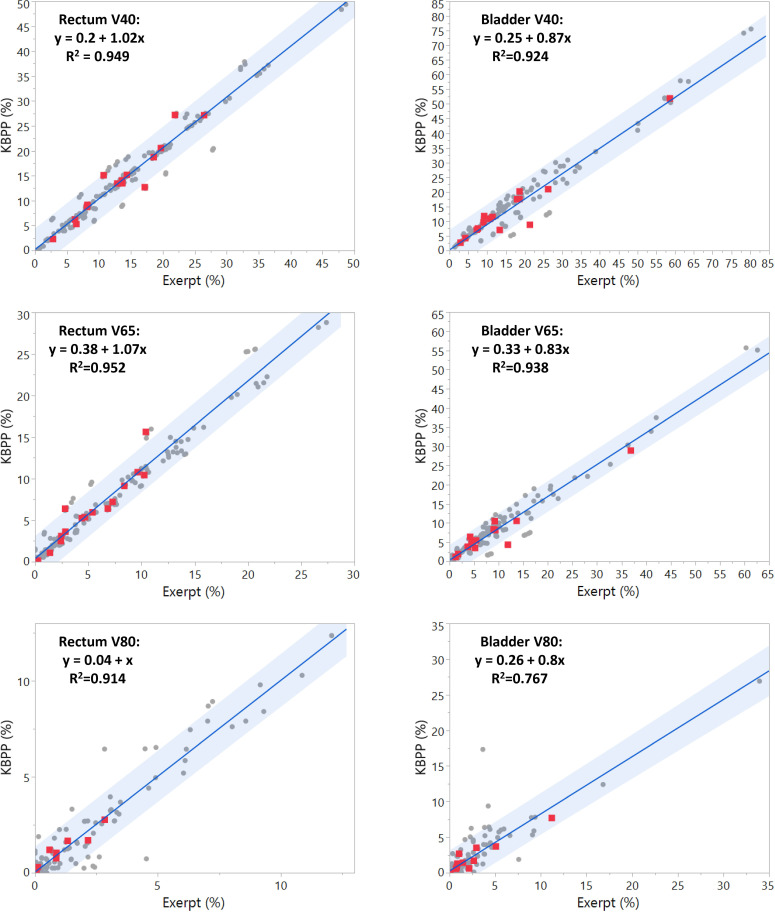
Dose-volume indices achieved by KBP versus expert for rectum and bladder for MFO-based plans. Red dots represent the dose-volume indices from nominal plans while gray dots represent the ones from other uncertainty scenarios. The blue band represents the confidence interval. Abbreviations: KBP, knowledge-based planning; MFO, multifield optimization.

**Figure 2** shows the scatter plots of selected dose-volume indices of the nominal (red squares) and uncertainty-scenario (gray dots) plans represented for the rectum and bladder. The dose-volume indices for rectum and bladder from KBPP plans shows good correlation with those achieved by the expert plans. The regression coefficients for V80Gy of rectum and bladder are relatively smaller (0.914 and 0.767, respectively), which is due to the small value of the V80Gy.

**Figure 3.**
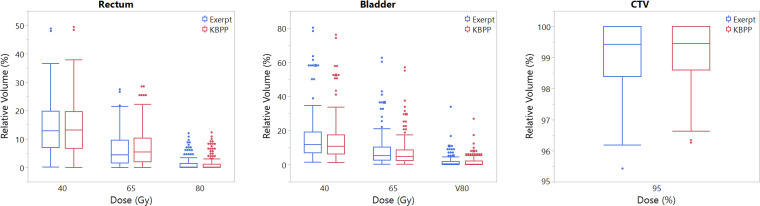
Box plots of dose-volume indices with all scenarios included for (a) rectum, (b) bladder and (c) CTV for MFO based-plans. Abbreviations: CTV, clinical target volume, MFO, multifield optimization.

**Figure 3a** and **3b** shows box plots of the same dose-volume indices for the rectum and bladder, which further confirms that KBP and expert plans are comparable in rectum and bladder sparing. **Figure 3c** is the box plot for CTV V95, demonstrating that KBPP plans are slightly more robust than the expert plans in CTV coverage.

**Figure 4.**
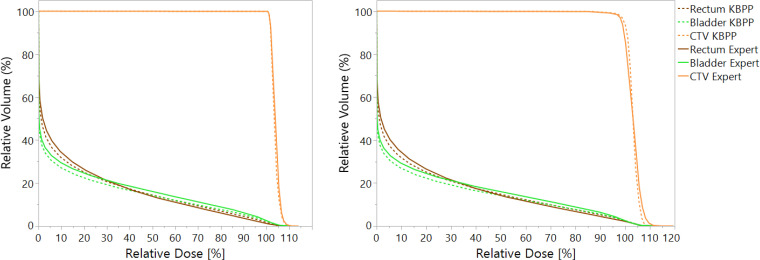
DVHs averaged over all 10 plans for KBPP and expert plans for nominal plans (a) and plans including all uncertainty scenarios (b) for MFO-based plans. Abbreviations: DVH, dose-volume histogram; KBPP, proton-specific knowledge-based planning; MFO, multifield optimization.

**Figure 4a** shows the average DVH of nominal plans, whereas **Figure 4b** consists of the average DVH, including all 13 scenarios (1 nominal plan plus 12 uncertainty scenarios). Almost no difference was observed between the KBPP and expert plans in the CTV DVH in **Figure 4a**. However, when all scenarios were included, it was obvious that the CTV curve from the KBPP plans had a sharper shoulder, reflecting that the KBPP generated more-homogenous plans.

**Figure 5.**
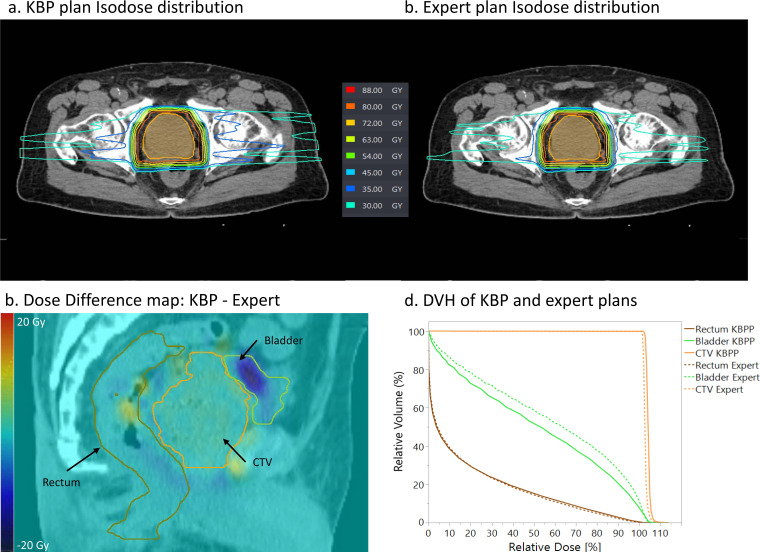
(a and b) Isodose distributions of KBPP and expert nominal plans using MFO technique for a prostate patient included in this study. (c) The dose difference map between KBPP and expert plan. (d) DVH for the KBPP and expert nominal plans. Abbreviations: DVH, dose-volume histogram; KBPP, proton-specific knowledge-based planning; MFO, multifield optimization.

**Figure 5** is an example of both the KBPP and the expert plans failing to meet the dose constraints for the bladder because of its extremely small volume. The expert plan exhibited slightly better dose distribution at low dose levels (**Figure 5a** and **5b**). Nevertheless, the KBPP plan, overall, accomplishes better bladder sparing. **Figure 5c** is a difference map of the KBPP and expert plan dose distributions. This image reveals that there is a 10 to 15 Gy dose difference between the 2 plans for the bladder region. A similar DVH curve for the rectum can be seen in **Figure 5d**. Comparable CTV coverage is achieved by KBPP and expert nominal plans, but the KBPP plan is more robust when assessing the V95 from the worst-case scenarios (CTV V95 = 98.23% versus 97.41% for KBPP and expert plans, respectively).

## Discussion

To our knowledge, this was the first study to explore the performance of the KBPP for robustly optimized IMPT in the treatment of prostate cancer. This work demonstrates that the IMPT plans generated by a KBPP model were able to achieve comparable plan quality to that of the IMPT plans generated by experts. This is consistent with the previous results on the use of the KBPP model for IMPT planning for head and neck cancers and hepatocellular carcinoma [20, 22, 23]. One of the benefits of employing the KBPP system for plan generation is its high efficiency. On average, each expert plan required 45 minutes to complete iterative optimization and dose calculation. In comparison, it took about 10 minutes for KBPP plan generation. Most KBPP plans were generated with a single optimization, whereas a subsequent “continue optimization” was performed for 2 cases, which took an additional 3 minutes. With respect to dosimetric indices, although the differences between the expert- and KBPP-generated plans were statistically significant for the dose volume indices for rectum V65Gy (0.8% ± 2.94%), it is likely that these differences are not clinically relevant because the magnitude of the differences was small. Statistically significant lower dose-volume indices for bladder were found in the KBPP plans compared with the expert plans. For an extreme case, for example, and extremely small bladder, the KBPP plan may produce better OAR sparing. The analysis that included all uncertainty scenarios indicated that the plans generated by the KBPP model were more robust and homogenous than the plans generated by the experts. Because the KBPP models accounts for robustness parameters, that increase in robustness was anticipated. When using this model for SFO-based plans generation, KBPP-generated plans also offered comparable plan quality to that of the expert SFO plans. We noticed that the SFO technique can provide more-robust and -homogenous plans in terms of CTV coverage compared with the MFO-based plans, but the MFO-based plans for localized prostate cancer were able to achieve robust coverage of the CTV, despite extreme rotational and translational alignment errors [28]. In SFO plans, we generally observed field-specific hot spots in the range of 55% to 60% of the total dose, and in the MFO plans, a typical field-specific hot spot was in the range of 65% to 70%, always from a beamlet near a bone junction; however, most of the dose distribution was evenly shared between beams. However, the MFO plans were able to provide greater volumetric coverage of the seminal vesicles for some anatomies. Because the MFO plans were able to provide satisfactory homogeneous coverage, we believe the extra degree of freedom in planning could be used for greater sparing in abnormal anatomies.

This study included 30 cases for model training without any outlier removal in our study. Outliers identified by the KBP system indicate that the plan has a statistically significant difference as compared with that for the entire population in the model. However, earlier studies by Delaney et al [27] and Hussein el al [15] compared the quality of the plans generated by an outlier-free model to a model without outlier removal, which demonstrated that the effect of a few outliers does not significantly affect plan quality [15, 27]. Our previous [29] also showed that the differences between the refined KBP model generated by eliminating the dosimetric outliers and the original KBP-generated plans were insignificant. It has been reported that, in the KBPP model for prostate cancer treatment, the initial automated-model generation setting led to inferior target coverage to that of the expert plans, indicating more refinement of the model was required [15, 30]. In our study, we implemented defined objectives for structures and let the model create the priority values for each objective. The KBPP plans produced by our model achieved high plan quality and even better CTV coverage compared with that of the expert plans. It was reported that in VMAT model training, the size of the models (33 versus 66 versus 97) made no difference in the plan quality for prostate cases [29]. In this work, a proton model that included 30 cases with all contours of bladder and rectum was reliable for the simple prostate IMPT plans.

In this work, we employed parallel-opposed lateral fields for both expert and KBPP generated robustly optimized IMPT plans. It has been shown that IMPT plans with 3 optimized beam angles may significantly improve rectum sparing compared with the conventional approach [31]. Furthermore, the quality of IMPT plans is more dependent on beam arrangement than photon plans are. It is worthwhile to explore the reliability of the KBPP model when plans with different beam arrangements are included in the model training. Future work on the integration of an algorithm with automated beam-angle selection, which is under investigation [32], to determine whether there can be further improvement in plan quality and efficiency.

Admittedly, the prostate is a quite-simple model to start such analyses and is good practice for the application of the KBPP model for robustly optimized IMPT plan creation. One limitation of this study is that we only included 15 patients for model validation, which may be insufficient to confirm the reliability of the model because such analyses are in an early stage for KBPP exploration. That said, many publications on KBP models have included small numbers of plans for validation, and vendor recommendations are 10 validation cases to prove the model is working sufficiently [20, 29]. Further work on more-complicated cases should be performed, and it is necessary that the model be validated before it can be implemented for clinical use.

## Conclusions

This work explored the reliability of a KBPP model to generate robustly optimized IMPT plans for patients with localized prostate cancer. The results demonstrated that the IMPT plans created by the model have high quality and are comparable the ones generated by experts. Furthermore, a KBPP model was able to generate more-robust and -homogenous plans than those of the expert plans. Use of the KBPP model for generation of IMPT plans has a potential to improve treatment-planning efficiency. More studies need to be performed for validation of the KBPP model for more-complicated treatment sites.
